# Evaluating the SERCA2 and VEGF mRNAs as Potential Molecular Biomarkers of the Onset and Progression in Huntington’s Disease

**DOI:** 10.1371/journal.pone.0125259

**Published:** 2015-04-27

**Authors:** Federica Cesca, Elisa Bregant, Borut Peterlin, Maja Zadel, Giorgia Dubsky de Wittenau, Gabriele Siciliano, Roberto Ceravolo, Lucia Petrozzi, Giada Pauletto, Lorenzo Verriello, Paolo Bergonzi, Giuseppe Damante, Giovanni Barillari, Bruno Lucci, Francesco Curcio, Incoronata Renata Lonigro

**Affiliations:** 1 Dipartimento di Scienze Mediche e Biologiche, Università degli Studi di Udine, Udine, Italy; 2 SOC Istituto di Genetica, Azienda Ospedaliero Universitaria di Udine, Udine, Italy; 3 Clinical Institute of Medical Genetics, University Medical Centre, Ljubljana, Slovenia; 4 Community Health Centre Ljubljana, Metelkov, Ljubljana, Slovenia; 5 Dipartimento di Medicina Clinica e Sperimentale, Università degli Studi di Pisa, Pisa, Italy; 6 Clinica Neurologica Azienda Ospedaliero Universitaria di Udine, Udine, Italy; 7 Medicina Trasfusionale, Azienda Ospedaliero Universitaria di Udine, Udine, Italy; 8 Neurologia, Azienda Ospedaliera, Santa Maria degli Angeli, Pordenone, Italy; 9 SOC Istituto di Patologia Clinica, Azienda Ospedaliero Universitaria di Udine, Udine, Italy; Inserm U837, FRANCE

## Abstract

Abnormalities of intracellular Ca^2+^ homeostasis and signalling as well as the down-regulation of neurotrophic factors in several areas of the central nervous system and in peripheral tissues are hallmarks of Huntington’s disease (HD). As there is no therapy for this hereditary, neurodegenerative fatal disease, further effort should be made to slow the progression of neurodegeneration in patients through the definition of early therapeutic interventions. For this purpose, molecular biomarker(s) for monitoring disease onset and/or progression and response to treatment need to be identified. In the attempt to contribute to the research of peripheral candidate biomarkers in HD, we adopted a multiplex real-time PCR approach to analyse the mRNA level of targeted genes involved in the control of cellular calcium homeostasis and in neuroprotection. For this purpose we recruited a total of 110 subjects possessing the HD mutation at different clinical stages of the disease and 54 sex- and age-matched controls. This study provides evidence of reduced transcript levels of sarco-endoplasmic reticulum-associated ATP2A2 calcium pump (SERCA2) and vascular endothelial growth factor (VEGF) in peripheral blood mononuclear cells (PBMCs) of manifest and pre-manifest HD subjects. Our results provide a potentially new candidate molecular biomarker for monitoring the progression of this disease and contribute to understanding some early events that might have a role in triggering cellular dysfunctions in HD.

## Introduction

Huntington's disease (HD) is a dominantly inherited neurodegenerative disorder involving brain and systemic alterations. The disease is characterized, among others, by the progressive loss of striatal medium spiny neurons, with an onset of symptoms at a mean age of 45 years. Progressive motor and cognitive decline and the development of psychiatric symptoms [1,2] are well known signs of the disease. HD is caused by the expansion of a polyglutamine stretch in the huntingtin (HTT) protein [3]. Mutant huntingtin (mHTT) gains toxic function [4–15], primarily in medium spiny neurons (MSNs) [16], with aberrant interactions in several pathways, including gene expression, oxidative stress, mitochondrial function, energy metabolism, Ca^2+^ homeostasis and signalling. mHTT is ubiquitously expressed and there is growing evidence that the mutant protein causes cytotoxicity not only in the central nervous system but also in the peripheral tissue of patients with HD [17]. Unfortunately, there is no known cure for this disease. To plan an early and effective therapeutic intervention, it is necessary to know the molecular mechanisms that trigger cellular dysfunction in HD, even in the period preceding the onset of symptoms. Furthermore, molecular biomarker(s) for the monitoring of disease onset and/or progression and response to treatment, need to be identified. However, the non-invasive detection of clinically useful biomarkers is a challenge faced by many research laboratories in the last few years. Multiple alterations in gene expression patterns, mainly using microarray technology and the further validation of selected genes, have been shown in the peripheral tissues of HD patients and in the peripheral blood of mouse models of HD [17–24]. For instance, variation in the expression level of genes involved in energy metabolism, oxidative stress and inflammatory response in peripheral leukocytes of HD patients with respect to control individuals have been reported [21–23]. Different studies have suggested that the toxic mHTT gain of function may involve disturbed glutamate-induced Ca^2+^ signalling in MSNs [9,25]. It has been reported that mHTT facilitates the activity of NMDA receptors [15,26–30] and type 1 inositol 1,4,5-triphosphate receptors (InsP_3_R1) [31,32], leading to enhanced intracellular Ca^2+^ concentrations in HD neurons. Furthermore, in peripheral tissues altered RNA expression of genes linked to Ca^2+^ homeostasis in HD patients with respect to controls has been reported (17). In spite of the fact that one of the hallmarks of HD is an impairment of the intracellular Ca^2+^ concentration and signalling, only recently the expression of genes encoding the calcium signalosome has been investigated in cellular and transgenic models of HD [33]. In this paper the authors describe changes in the gene expression of members of the calcium signalosome. However, these changes differ in the two models of HD used in the study. To date, the gene expression of calcium pumps dedicated to the extrusion of calcium from the cytoplasm (PMCA1-4 isoforms) or its storage in the cisterns of the endoplasmic reticulum (SERCA pumps) has not yet been directly explored. In this work, we investigated the mRNA levels of both the housekeeping-expressed plasma membrane-associated PMCA1 isoform [34–35] and the sarco-endoplasmic reticulum-associated (SERCA2 and SERCA3) pumps in PBMCs from manifest and pre-manifest HD patients compared to sex- and age-matched controls.

Furthermore, to date, the expression level of neurotrophic and neuroprotective agents as potential tracer(s) of disease onset and/or progression has not been sufficiently explored. Indeed, neurotrophic factors such as brain-derived neurotrophic factor, glial-derived neurotrophic factor and ciliary neurotrophic factor are transcriptionally deregulated in HD striatal neurons and have been found to be neuroprotective in a variety of animal models of HD [18–20,36,37]. Among others, vascular endothelial growth factor (VEGF) has neurotrophic and neuroprotective properties in addition to its well-known angiogenic functions. VEGF directly exerts neuroprotective actions, through the inhibition of programmed cell death or apoptosis and the stimulation of neurogenesis [38–45], as well as indirectly, by activating antioxidants, promoting angiogenesis and enhancing blood brain barrier permeability for glucose [46–53]. Although VEGF acts as an endogenous neuroprotective factor, it is produced by both neuronal and non-neuronal cells, including endothelia and astroglia. VEGF released from these sources might target VEGF receptors on the surface of neurons [40] and contribute to their tropism and survival.

In this study, we also investigated the level of VEGF mRNA expression in PBMCs from the above-described cohort of HD and control subjects. Our results provide a potentially new molecular biomarker for monitoring the progression of the disease and shed new light on some early events that may contribute to cellular dysfunction in HD.

## Materials and Methods

### Human ethics

All samples were obtained in accordance with the institutional Bioethics Committee named “*Comitato Etico dell’Azienda Ospedaliero-Universitaria Santa Maria della Misericordia*”, which approved this study. The patients or their legal guardians gave written informed consent.

### Patients recruitment and samples collection

All the patients and controls were residents of Italy and Slovenia. The HD group consisted of 90 HD manifest patients, of which 45 were male and 45 female (average age 54.1 ± 12.8 years), and 20 HD pre-manifest patients, 10 male and 10 female (average age 38.8 ± 8.4 years). The control group consisted of 54 healthy subjects (33 male and 22 female) matched for sex and age, who were recruited among blood donors and without a family history of HD. All samples were collected between 9,00 and 10,00 a.m. in fasting conditions and a sample (5 ml) of peripheral blood was collected in Vacutainer tubes with EDTA and processed with FicollPaque (GE Healthcare) according to the manufacturer’s instructions. Peripheral blood mononuclear cells (PBMCs) were collected.

### Clinical classification of patients

All the subjects, patients and controls, were grouped arbitrary in three range of age (27–40; 43–58; 59–84 years old; range of CAG repeats 47–57, 49–56 and 37–49 respectively) in an attempt to account for aging-dependent variation of gene expression. Alternatively, where indicated, the HD patients enrolled in this study were stratified upon their neurological status. The Unified Huntington’s Disease Rating Scale (UHDRS) by an experienced HD neurologist has been used to determine neurological status. All of the manifest patients were in stages I or II of HD as determined by total functional capacity scores of 7–12. UHDRS total motor score (UHDRS-TMS) between 5 and 24 defined early-manifest patients, UHDRS-TMS between 25 and 50 defined manifest patients, and a score of more than 50 defined late-manifest HD patients. In summary, the 20 pre-manifest patients (mean age 40.9 ± 5.5 years), relatives of already manifest subjects, were carriers of the HD-causing mutation (range of CAG repeats 39–48) but still negative by clinical evaluation; the 17 early-manifest patients (mean age 47.9 ± 10.5; range of CAG repeats 40–52) experienced the onset of poor control of voluntary movements of the limbs and facial grimaces with or without psychic disorders; the 18 late-manifest patients (mean age 59.3 ± 11.9 years; range of CAG repeats 40–57) usually presented dementia as well as marked hyperkinesis or even severe spastic tetraplegia and mutism. The remaining 55 subjects (mean age 52.2 ± 11.1 years; range of CAG repeats 37–50) were grouped in the manifest category.

No patients had any medication suspected or known to interfere with the experimental methods used and/or with the gene expressions analysed.

### DNA extraction and molecular diagnosis of HD

DNA was isolated using the *Purgene DNA Purification kit* (Gentra Systems) according to the manufacturer’s instructions. The DNA concentration was determined using the *Quantifluor dsDNA System* (Promega), and the DNA quality was determined on a 1% agarose gel (TBE 0.5 X) with GelRed (Biotium) using *Lambda DNA/HindIII* as a marker of molecular weight (Fermentas).

To evaluate the number of CAG repeats and to exclude false homozygous states that can be due to the occurrence of SNPs in the region of primer annealing, we performed three independent PCR reactions. Briefly, the primers HD1F and HD3R were used to amplify the CAG repeat, HD4 and HD5 were used to amplify the CCG repeat immediately downstream of the CAG region, and HD1 and HD5 were used to amplify the entire CAGCCG region. The sequences of the primers and references are shown in Table 1. The protocol of the PCR reaction mix and cycles was optimized to be unique in getting separate amplicons of the three repeated regions. Precisely, the PCR reactions were performed in a final volume of 25 μl containing 4 μl of each specific primer (5 pmol/μl), 2.5 μl of buffer 10X (Applied Biosystem), 7.5 μl glycerol 50%, 0.88 μl dimethyl-formamide, 1.0 μl dNTPs (5 mM), 0.38 μl AmpliTaq DNA polymerase (5 U/μl) (Applied Biosystems), 2 μl of genomic DNA (100 ng/μl) previously denaturated at 95°C for 7 min and water to 25 μl. The amplification was performed with an initial denaturation at 94°C for 1 min, followed by 35 cycles of denaturation at 94°C for 30 sec, annealing at 66°C for 30 sec and elongation at 72°C for 30 sec and a final extension at 72°C for 7 min. The PCR fragments were resolved by capillary electrophoresis on an automated ABI Prim 3730 XL Genetic Analyzer in presence of HiDi formamide and Gene Scan 500-LIZ 500 internal size standard (Applied Biosystems). The data were analysed using Peak Scanner Software (Life Technologies).

**Table 1:** Primers used to evaluate the number of CAG repeats for the molecular diagnosis of HD.

Name	Sequence	Length	Amplified Region	Reference
HD1F	ATGAAGGCCTTCGAGTCCCTAAAGTCCTTC	30 bp	CAG	[54]
HD3R	GGCGGTGGCGGCTGTTGCTGCTGCTGCTGC	30 bp		
HD4F	GCAGCAGCAGCAGCAACAGCCGCCACCGCC	30 bp	CCG	[55]
HD5R	GCGGCGGCTGAGGAAGCTGAG	21 bp		

### RNA preparation and reverse transcription

Total RNA from PBMCs was extracted with *RNeasy Mini Kit* (Qiagen) according to the manufacturer’s instructions, and 450 ng of total RNA was reverse-transcribed using *SuperScript III Reverse Transcriptase* (Life Technologies) and random hexaprimers (Invitrogen), according to the manufacturer’s instructions. Three independent reactions were performed for each RNA sample to correct for inter-assay variability. Briefly, the RNA plus random hexaprimers was rapidly denatured at 65°C for 5 min, and the RT-PCR protocol was carried out as follows: 55°C for 60 min, 70°C for 15 min and 95°C for 5 min, followed by cooling at 4°C. The three RT-PCR products, relative to each RNA sample, were pooled and used in three independent real-time PCR assays.

### Relative RNA quantification

For the detection and relative quantification of target gene transcripts, TAQMan gene expression assays (Applied Biosystems) and a 7500 Real-Time PCR System (Applied Biosystems) were used. β-actin was used as the reference gene. Each target and reference gene were co-amplified in the same reaction tube to minimise variability. The list of human assays for target genes is shown in Table 2. The sequences of the primers and probe used for β-actin are reported in Table 3. Probes specific for the target genes were labelled with the FAM fluorophore, and the β-actin probe was labelled with the VIC fluorophore. Each duplex real-time PCR contained 6.5 μl of 2X MasterMix (Applied Biosystems), 0.3 μl of gene-specific TAQMan gene expression assay, 0.55 μl of β-actin forward primer (10 μM), 0.55 μl of β-actin reverse primer (10 μM), 0.125 μl of β-actin probe (10 μM), 0.5 μl 50 mM MgCl_2_, 2 μl cDNA template and water to 12.5 μl. In all the assays the gene expression levels detected in the samples tested have been reported to the gene expression levels detected in the ARO cell line that was used as an internal calibrator sample.

**Table 2:** List of genes assessed for their expression level using multiplex real-time PCR assays and annotation.

Gene name/symbol	Description	Applied Biosystems assay ID
ATP2B1/PMCA1	ATPase, Ca^++^ transporting, plasma membrane 1	Hs00155949_m1
ATP2A3/SERCA3	ATPase, Ca^++^ transporting, ubiquitous	Hs00193090_m1
ATP2A2/SERCA2	ATPase, Ca^++^ transporting, cardiac muscle, slow twitch 2	Hs00544877_m1
VEGF	Vascular endothelial growth factor A	Hs00900054_m1

**Table 3:** Primer and probe sequences used for the real-time detection of β-actin.

Name	Sequence	Length
β -actin 611 (TAMRA probe)	ACC ACC ACG GCC GAG CGG	18 bp
β -actin-592F (forward primer)	CGA GCG CGG CTA CAG CTT	18 bp
β -actin-651R (reverse primer)	TCC TTA ATG TCA CGC ACG ATT T	22 bp

### Data processing and statistical analysis

Experimental data from real-time PCR were reported as threshold cycle (Ct) values and summarized as mean ± SD of three independent experiments, as indicated in the figure legends. Each experiment was performed on individual samples in triplicate. Ct values for each gene were normalized to internal control (β-actin) that was run with each tested sample. Differences in gene expression were calculated using the ΔΔCt method, as previously described [56]. The significance of expression differences between tested groups was calculated using a two-tailed unpaired Student’s t-test, for the comparison of two means, by the QuickCalcs on-line software (www.graphpad.com) and p values <0.05 were considered to be significant. Linear regression analysis was employed to assess correlation between number of CAG repeats and gene expression levels.

## Results

### Probing RNA level of PMCA1, SERCA2, SERCA3 and VEGF in peripheral blood of a limited cohort of HD patients and controls

**Fig 1:**
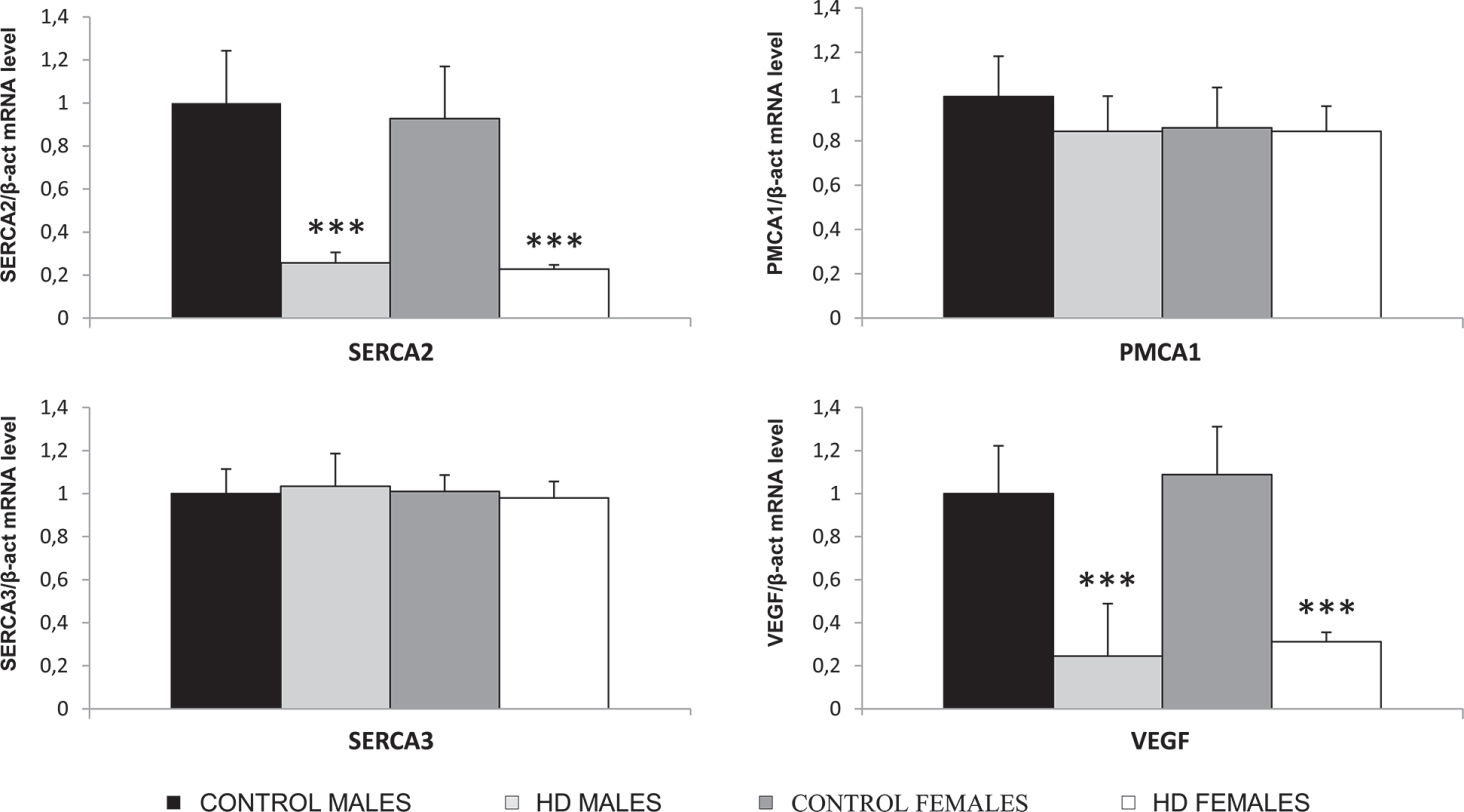
RNA quantification of PMCA1, SERCA2, SERCA3 and VEGF in PBMCs from healthy controls and HD patients divided according to sex. Subjects analysed: 10 healthy male controls and 10 healthy female controls age-matched ± 3 years; 10 males HD, mean age of 44.5 ± 10.1; 10 females HD, mean age of 53.4 ± 9.6. *** p<0.005. Each column represents the relative mean value of three independent experiments ± SD.

To address the steady-state level of PMCA1, SERCA2-3 and VEGF transcripts in the peripheral blood of HD patients and controls, RNA was extracted from PBMCs of 20 HD subjects (10 males, with a mean age of 44.5 ± 10.1, and 10 females, with a mean age of 53.4 ± 9.6) and 20 age-matched (± 3 years) and sex-matched (10 males and 10 females) healthy controls. Each RNA was reverse transcribed in three independent reactions, and 2 μl of pooled cDNA was probed for the evaluation of the gene-specific expression level, as described in Materials and Methods. As shown in Fig 1, the mRNAs of PMCA1 and SERCA3 showed similar levels in the HD and control groups, both in the male and female subjects. In contrast, the levels of SERCA2 and VEGF mRNAs were significantly reduced in the HD patients (p<0.005) compared to the age- and sex-matched controls. Because no sex-related differences were found regarding the mRNA level of the four genes tested, all further analyses were performed pooling the results obtained from males and females for both the HD and control subjects.

### The mRNA level of SERCA2 is reduced in HD manifest as well as pre-manifest subjects and has a negative correlation with the expansion of the CAG repeat

**Fig 2:**
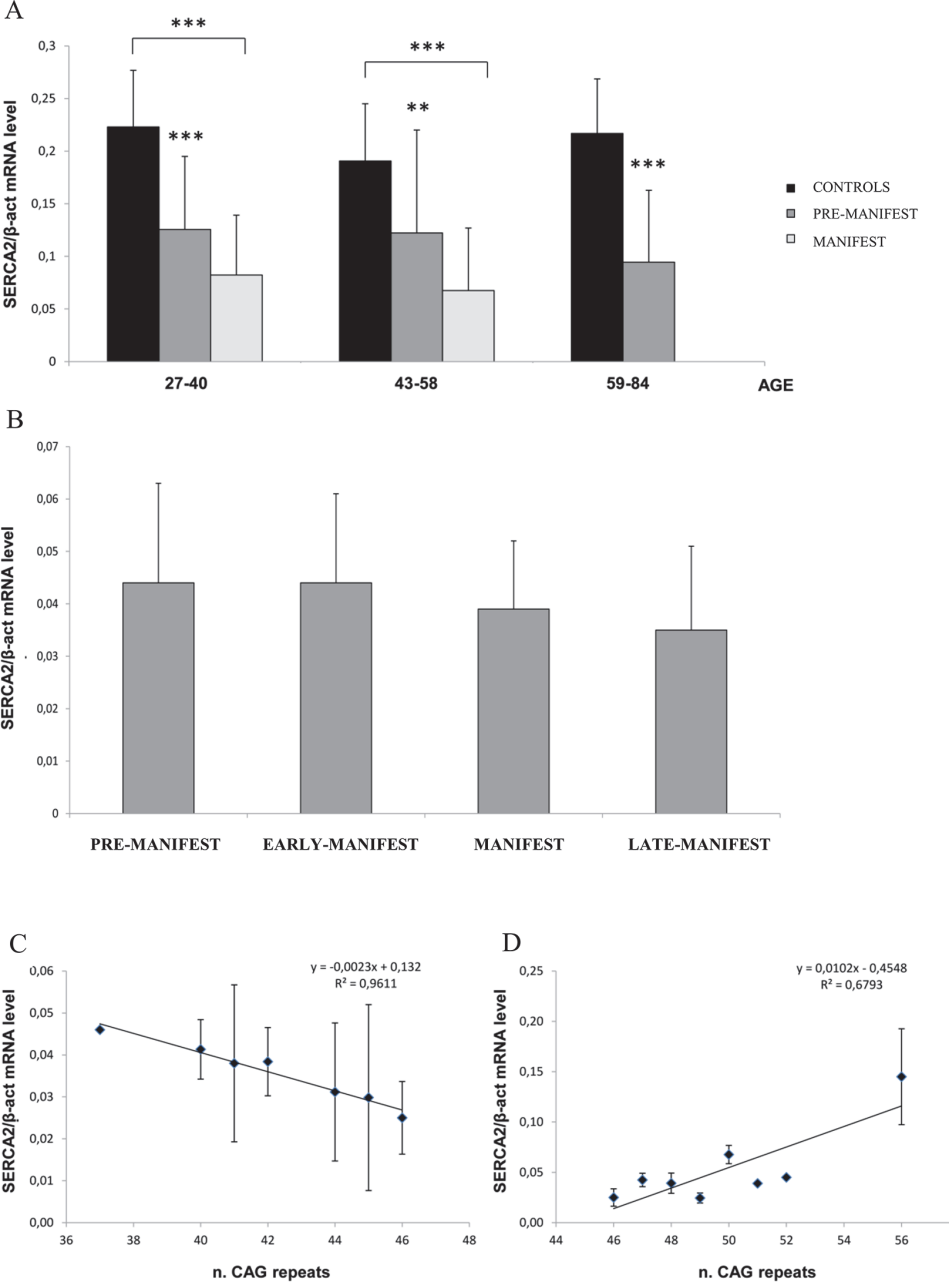
mRNA quantification of the SERCA2 pump relative to the age, clinical conditions and CAG expansion of the patients. (A) Comparison of the level of SERCA2 mRNA in PBMCs from HD pre-manifest and manifest patients and age- and sex-matched healthy controls. Age 27–40: Controls = 15; pre-manifest = 12; manifest = 13; Age 43–58: Controls = 26; pre-manifest = 8; manifest = 42; Age 59–84: Controls = 13; manifest = 35. ** p<0.01; *** p<0.001. (B) Comparison of the level of SERCA2 mRNA in PBMCs of patients with different stages of the disease: pre-manifest = 20; early-manifest = 17; manifest = 55; late-manifest = 18. (C) Correlation between the level of SERCA2 mRNA and the number of CAG repeats (range 37–46) R^2^ = 0.9611. (D) Correlation between the level of SERCA2 mRNA and the number of CAG repeats (range 46–56) R^2^ = 0.6793. The mean values ± SD of three independent assays are shown.

The steady-state level of SERCA2 mRNA in PBMCs was analysed on a total of 164 subjects comprised of Italian and Slovenian individuals. In total, 90 HD manifest patients (45 males and 45 females), 20 pre-manifest carriers of HD causative mutation (10 males and 10 females) and 54 age- and sex-matched controls (33 males and 22 females) were analysed. Because SERCA2 gene expression has been reported to be reduced with ageing [57–59], the subjects analysed were subdivided in three arbitrary age ranges: 27–40, 43–58 and 59–84 years. No pre-manifest subjects were available in the 59–84 range of age. As shown in Fig 2A, all the HD-affected patients and even all the pre-manifest subjects displayed a highly significant reduction in SERCA2 mRNA, ranging from p<0.01 and p<0.001, compared with the control mRNA levels. However, the reduction of SERCA2 mRNA in the manifest versus pre-manifest subjects did not reach statistical significance. Furthermore, as shown in Fig 2B, no progressive reduction of SERCA2 mRNA was observed when stratifying the patients according to their clinical phenotype, namely, pre-manifest (N = 20), early-manifest (N = 17), manifest (N = 55) and late-manifest (N = 18). This result suggests that the impairment in SERCA2 gene expression is not a biomarker of onset and progression of the disease, however it is a precocious sign of events underlying cellular dysfunction in HD. Interestingly, by plotting the SERCA2 mRNA level against the number of CAG repeats, in the range of 37 and 46 CAG (Fig 2C), a linear regression of R^2^ = 0.9611 was observed. No further reduction in SERCA2 RNA was detected for the 25 HD subjects with CAG expansion greater than 46 repeats, from 47 up to 56 CAG (Fig 2D). In an attempt to ensure cell survival, some compensatory mechanisms may be activated when a higher CAG expansion is present. Conversely, specific molecular interactions, which may be responsible for the observed reduction in SERCA2 mRNA, may occur in the case of lower expansion (37–46 CAG repeats). Further investigation needs to be performed to understand the molecular mechanisms that inversely correlate SERCA2 mRNA expression and CAG repeats (i.e., 37–46). Some hypotheses on possible mechanisms underlying this observation will be discussed.

### The mRNA level of VEGF is more reduced in HD manifest than in pre-manifest patients with respect to controls, and no correlation is evident with the expansion of the CAG repeat

**Fig 3:**
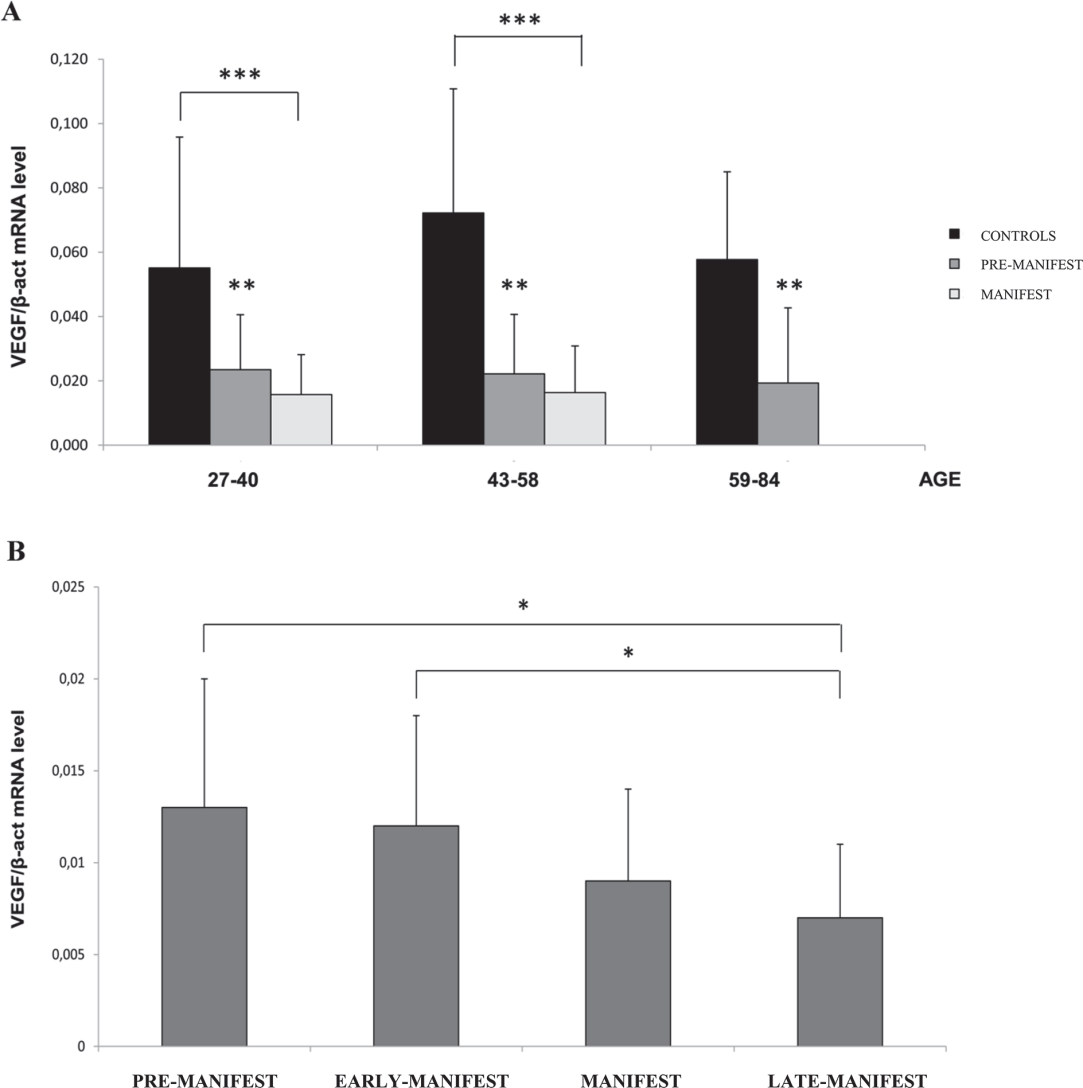
mRNA quantification of VEGF relative to the age and clinical conditions of the patients. (A) Comparison of the level of VEGF mRNA in PBMCs from HD pre-manifest, manifest patients and age- and sex-matched healthy controls.Age 27–40: Controls = 15; pre-manifest = 12; manifest = 13; Age 43–58: Controls = 26; pre-manifest = 8; manifest = 42; Age 59–84: Controls = 13; manifest = 35; ** p<0.01; *** p<0.001. (B) Comparison of the level of VEGF mRNA in PBMCs of patients with different stages of the disease: pre-manifest = 20; early-manifest = 17; manifest = 55; late-manifest = 18;* p<0.05. The mean values ± SD of three independent assays are shown.

The results obtained by analysing the steady-state levels of VEGF mRNA in the PBMCs of the same 164 subjects are shown in Fig 3A. As found for SERCA2 mRNA, the VEGF mRNA levels were also lower in all subjects carrying the mutation, both the pre-manifest (p<0.01) and manifest (p<0.001) subjects, when compared to the sex- and age-matched controls. Moreover, the mRNA level appeared to be inversely correlated with the onset and progression of the disease. Indeed, as shown in Fig 3B, by stratifying the patients according to their clinical phenotype, a significant reduction in VEGF mRNA (p<0.05) was observed in the late-manifest compared to the early and pre-manifest patients. Although this observation needs to be refined through the analysis of a greater number of subjects, it suggests a potential role of VEGF as a candidate biomarker useful for tracing the progression of Huntington's disease. No correlation was observed between the level of VEGF mRNA and the number of CAG repeats in any range analysed (data not shown).

## Discussion

The identification of quantitative biomarkers of neurodegeneration that highlights the onset and/or progression of symptoms remains a priority for patient management and for developing effective therapies. Huntington’s disease remains an incurable and fatal disease due to a toxic gain of function by the HTT mutant protein. Recent evidences show that mHTT induces alterations in areas of the central nervous system as well as in the peripheral tissues of patients with HD. The availability of peripheral biomarkers for tracing the onset and/or progression of the disease represents a reasonable goal for the clinical management of HD patients. Within this context, the number of studies aiming to identify molecular alterations in the peripheral tissues of patients and/or animal models of HD is increasing. Different attempts to compare gene expression profiles of HD tissues and matched controls have been made using microarray technology. Borovecky et al. [18] performed a genome-wide expression profiling of human blood and showed, in pre-manifest and manifest HD subjects, a significant up-regulation of genes belonging to many different functional groups, such as transcription / RNA processing, ubiquitin / proteasome, vesicle trafficking, and signal transduction, as compared to control goups. The up-regulation of twelve selected genes has been confirmed through qRT-PCR assays. However, these promising results were not reproduced by Runne and co-workers [19] on a different cohort of HD patients and controls. Nor other potential useful marker has been found by the peripheral transcriptomic analysis performed by these authors. This suggests that peripheral transcriptomics fails in providing common validated biomarkers in HD or rather emphasizes a need for further validations on new and enlarged cohorts of patients. Furthermore, differences in array platforms sensitivity and probes design and/or qRT-PCR detection methods and reagents and/or samples preparation methods may account for differences reported in different studies on gene expression profile in blood. Recently, in the attempt to find a cell type alternative to blood cells for biomarker studies in HD, Marchina et al. (17) performed gene expression profiling analysis on skin fibroblasts of HD patients and controls. Interestingly, in a set of nine genes confirmed to be differentially expressed between HD and controls the phosphoinositide-specific phospholipase C β4 (PLCβ4) gene is strongly upregulated. PLCβ4 over-expression could be linked to the altered Ca^2+^ homeostasis in this cell type in HD. Deregulated Ca^2+^ homeostasis is a well-established hallmark of HD. Considering the harmful effects of increased levels of intracellular calcium, the expression and activity of pumps able to actively counteract the augment of Ca^2+^ concentration in HD neurons may play a key role in the onset and/or progression of the disease. Plasma membrane and sarco/endoplasmic Ca^2+^ATPases, namely PMCAs and SERCAs pumps, play a pivotal role in cellular calcium homeostasis and in regulating the physiological resting level of calcium [60–61]. In our study, we adopted a multiplex real-time PCR approach to compare the RNA levels of the housekeeping-expressed PMCA1 pump and the SERCA2 and SERCA3 pumps in PBMCs isolated from blood samples of patients with HD, pre-manifest subjects and sex- and age-matched healthy controls. Comparable levels of PMCA1 and SERCA3 mRNAs were found in all HD and control groups examined, whereas the SERCA2 mRNA level was significantly reduced in all the subjects carrying the HD causative mutation, both manifest and pre-manifest subjects. These results suggest that, at least in the cohort of patients we examined, the impairment of SERCA2 gene expression is an early pathogenetic event underlying peripheral cells dysfunction in HD. However, because no significant and progressive reduction was observed by comparing SERCA2 mRNA expression levels in PBMCs from pre-manifest, early-manifest and late-manifest patients, SERCA2 could be considered as a biomarker of the genetic status but not of the onset or progression of the disease.

It must be said that apparently, the reduction of SERCA2 mRNA level in blood of HD patient respect to controls is not confirmed by the work of Diamanti et al. (24) on mice models of HD. Indeed, in the arrays analysis conducted by these authors on peripheral blood of R6/2 mice in the symptomatic stage and wild type littermate mice, even if some of the gene set arrays contain SERCA2-specific probes no altered expression profile of SERCA2 mRNA has been reported in blood of HD with respect to control mice. A simple explanation of these conflicting results could be that the microarray assays have a different sensitivity than the QPCR assays. Alternatively, an intriguing explanation could arise from the observation that the R6/2 mice express a short fragment of mHTT but carrying a large poly-Q trait (about 110Q). None of the patients we examined have a similar number of polyQ in the mHTT. Furthermore, as shown in Fig 2C, we found a significant inverse correlation between the expanded poly-Q tract of mHTT and the SERCA2 mRNA level only in the range of 37Q-46Q-46QQ. Further expansions of poly-Q trait (47QQ-56Q) are not accompanied by a further reduction of SERCA2 mRNA level in blood, rather an opposite finding, though not significant in that group of patients, seems to exist with more than 46Q (Fig 2D). This observation suggests that peculiar molecular interaction(s) could link the mHTT protein and SERCA2 gene expression in a polyQ-dependent manner. One possible explanation of this could be that different conformational/functional properties are acquired by the mutant proteins with differently expanded polyQ traits, and this could affect a gene-specific expression. The first unveiled mechanism drawing transcriptional alterations in HD was a direct protein-protein interaction of mHTT with transcription factors. However, a more likely scenario has been depicted by Benn and co-workers [62] in demonstrating that the wild type and the mutant HTT both bind directly DNA, have different genomic binding sites in vivo, including different gene promoters and sites for transcription factor occupancy. The mHTT could inhibit a proper binding of transcription factors to DNA and/or alter DNA conformation and hence alter the gene transcription. These findings suggest the need of a more detailed investigation of the gene expression patterns related to a defined expansion of the poly-Q tract in HD. This is reinforced by our finding showing the occurrence of a progressive reduction of SERCA2 mRNA level only in a defined range of the poly-Q dimension. Clarifying the peculiar molecular interaction(s) linking the mutant protein and SERCA2 gene expression could provide new insights in differential and early occurring molecular mechanisms that contribute to trigger cellular dysfunction in HD.

Another well-established hallmark of HD is the reduced expression of neurotrophic and neuroprotective agents. VEGF gene expression has also been found to be impaired in both manifest and pre-manifest patients, highlighting the need to improve the signalling of neuroprotective agents in HD. Interestingly, Ellison and co-workers recently exploited a lentiviral-mediated delivery of VEGF and 82Q mHTT in rat primary striatum cell cultures and in adult rat striatum, demonstrating a strictly dose-dependent neuroprotection exerted by the VEGF_165_ isoform in these models of HD [37]. In our study, the level of VEGF mRNA in PBMCs of HD mutation carriers was found to be inversely correlated with the progression of the disease. Indeed, when stratifying the patients according to their clinical phenotype, a significant reduction of VEGF mRNA was observed in blood of late manifest compared to early and pre-manifest patients. Although this observation needs to be confirmed through the analysis of a larger number of subjects and on different populations, it suggests a potential role of VEGF mRNA as a useful candidate biomarker for following up the progression of Huntington's disease.

In conclusion, to the best of our knowledge, data reported here are the only directly addressing the Calcium pumps and VEGF mRNA levels in blood of HD. Certainly the work we have presented has several limitations such as to validate the results obtained on a different cohort of patients and investigate the possible pathogenetic significance of the reduction of SERCA2 and VEGF mRNAs both in peripheral tissues and the central nervous system of HD patients. Consistent with the above, the evaluation of SERCA2 and VEGF protein levels in the peripheral blood of patients and controls is missing. Nevertheless, the data presented are consistent with the aim of this work that is the search for biomarkers in peripheral blood of HD patients useful in monitoring the onset and / or progression of the disease. Our overall results suggest that the evaluation over time of VEGF mRNA level in blood of HD patients could be helpful in monitoring the progression of the disease. Moreover, the evaluation of VEGF and SERCA2 mRNAs both could be helpful in monitoring the response to therapy in clinical trials. Finally, the results of our study could provide new insight to understand early events that could contribute to cellular dysfunction in HD.
